# Stereotactic Radiation for Treating Primary and Metastatic Neoplasms of the Spinal Cord

**DOI:** 10.3389/fonc.2020.00907

**Published:** 2020-06-09

**Authors:** Elisa K. Liu, Joshua S. Silverman, Erik P. Sulman

**Affiliations:** ^1^Departments of Radiation Oncology, NYU Grossman School of Medicine, New York, NY, United States; ^2^Departments of Neurosurgery, NYU Grossman School of Medicine, New York, NY, United States; ^3^Brain and Spine Tumor Center, Laura and Isaac Perlmutter Cancer Center, NYU Langone Health, New York, NY, United States

**Keywords:** radiotherapy, radiosurgery, spinal cord tumor, neoplasms, intradural, intramedullary

## Abstract

Stereotactic radiation treatment can be used to treat spinal cord neoplasms in patients with either unresectable lesions or residual disease after surgical resection. While treatment guidelines have been suggested for epidural lesions, the utility of stereotactic radiation for intradural and intramedullary malignancies is still debated. Prior reports have suggested that stereotactic radiation approaches can be used for effective tumor control and symptom management. Treatment-related toxicity has been documented in rare subsets of patients, though the incidences of injury are not directly correlated with higher radiation doses. Further studies are needed to assess the factors that influence the risk of radiation-induced myelopathy when treating spinal cord neoplasms with stereotactic radiation, which can include, but may not be limited to, maximum dose, dose-fractionation, irradiated volume, tumor location, histology and treatment history. This review will discuss evidence for current treatment approaches.

## Introduction

Intradural lesions, which can be further divided into extramedullary (IDEM) and intramedullary (IM), account for 30% of all spinal cord neoplasms (1). Surgery is the preferred method for symptom relief and tumor control. The safety and efficacy of microsurgical techniques have been well-documented for a variety of spinal cord lesions (1–5). In some situations, surgery is contraindicated by medical comorbidities, performance status, lesion location, or rapidly recurrent/progressive tumor. In addition, gross total resection (GTR), which has been shown to drastically improve tumor outcomes, may not be attainable in all cases (6, 7). Furthermore, studies on the efficacy of chemotherapy are limited but show no effect on survival (8). In cases where GTR is unachievable, stereotactic radiotherapy (SRT), or, when treatment is delivered in a single dose, stereotactic radiosurgery (SRS) may be important clinical tools for symptom management and tumor control.

Conventional radiation approaches typically rely on a standard dose fraction size of 180–200 cGy (9). While dose escalation attempts to 50 Gy or higher have led to improved control rates, the use of conventionally fractionated radiotherapy for both bony and spinal cord disease is associated with modest outcomes and may be additionally limited by the presence of multiple tumors or disease near organs-at-risk (10–12). Increasingly, treatment of the bony spine, largely metastatic disease, has shifted to using larger fraction sizes, or hypofractionation (dose >2 Gy), that provide potential benefit in improving tumor control and reducing the duration of treatment (13). Due to the large dose of radiation being delivered daily, hypofractionated approaches typically rely on stereotactic immobilization, such as body cradles, and real-time image guidance, such as cone-beam CT or orthogonal X-rays (14). While adoption of stereotactic radiation to treat the bony spine has increased, application of this type of treatment to spinal cord tumors is far less standardized or accepted.

Indeed, the utility of stereotactic radiation for treating spinal cord neoplasms is not well-defined. Most approaches are extrapolated from the success of treating intracranial tumors with SRT or SRS (15–17). However, the spinal cord is a serially organized tissue that may have its own unique radiobiological response to treatment (18). Furthermore, spinal cord lesions have unique presentations and close spatial relationships with the spinal cord, complicating the efficacy and safety of using stereotactic approaches (19). The goal of this review is to summarize existing experiences of treating IDEM and IM lesions with SRT and to highlight potential factors that influence radiation-injury.

## Methods

We conducted a literature search using the US National Library of Medicine PubMed database to identify English-language reports of intradural and intramedullary neoplasms treated using SRT or SRS. Reports containing a combination of the following terms were compiled: SRT, stereotactic radiotherapy, SRS, radiosurgery, intradural, intramedullary, extramedullary, tumors, metastases, and spinal cord. In addition, we employed a manual search strategy to review the references cited in order to identify further relevant studies.

## Results

### Metastatic Tumors of the Spinal Cord

Spinal metastases are seen in 40% of patients with cancer and are often associated with cerebrospinal fluid (CSF) dissemination, portending poor prognosis. Of all metastatic spinal disease, <6% are purely intradural. IM metastases are even more rare and are found in fewer than 1% of patients with systemic cancer (20).

Published reports of IDEM and IM metastases arise from a similar set of solid tumors, such as lung, breast, and renal cell carcinoma (21). IDEM metastases are additionally associated with central nervous system (CNS) drop-metastases in the pediatric population or in populations with primary brain malignancies (22). Patients often present with pain and neurological deficits that require more urgent intervention. This is in contrast to primary tumors which are often benign and have a longer duration of symptoms of less severity before intervention is sought (23). For both IDEM and IM metastases, SRT and SRS have been demonstrated to offer symptomatic control with minimal toxicities (Table 1).

**Table 1:** Studies on SRS for metastatic tumors of the intradural-extramedullary and intramedullary spinal cord.

**References**	**No. of pts/No. of lesions**	**No. of IDEM/No. of IM**	**Histology**	**Treatment details**	**F/u time**	**Outcomes**	**Complications**
Shin et al. (24)	9/10	4/6	Breast (3) lung (2), brain (2), kidney (1), skin (1)	Median 14 Gy (10–16 Gy)	10 mo	80% symptom improvement	None
						89% tumor control	
						mOS = 8 mo (range 2–19 mo)	
Mori et al. (25)	2/3	3/0	Lung (2)	Median 25 Gy in 5 frac (5–10 frac)	–	Partial improvement in symptoms	None
						Local control for treated lesions	
						mOS = 5 mo (range 5–22 mo)	
Veeravagu et al. (26)	9/11	0/11	Breast (5), lung (3), epitheloid hemangioepithilioma (1), teratoma (1)	Median 21 Gy (range 14–27 Gy) in 3 frac (1–5 frac)	–	Stable symptoms after treatment	None
						Local control for all lesions	
						mOS = 4.1 mo (range 1–9 mo)	
Parikh and Heron (27)	1/1	0/1	Kidney (1)	16 Gy in 3 frac	26 mo	Partial improvement in symptoms	None
						Tumor partial response	
						OS = 26 mo	
Lieberson et al. (28)	1/1	0/1	Prostate (1)	Marginal dose 27 Gy in 3 frac	3 mo	Improvement in symptoms	None
						Tumor controlled at f/u	
Garcia et al. (29)	1/1	0/1	Breast (1)	14 Gy in 1 frac	37 mo	Improvement in symptoms	Imaging showed asymptomatic radiation necrosis
						Tumor controlled at f/u	

*IDEM, intradural-extramedullary; IM, intramedullary; f/u, follow-up; frac, fractions; mo, months; mOS, median overall survival; OS, overall survival*.

Reports of IDEM metastases treated with SRT or SRS are limited to a small number of case series. Shin et al. reported four IDEM lesions treated with a single fraction of SRS, delivering either 14 or 16 Gy (24). Three of the four lesions had both clinical and radiographic responses, demonstrating no toxicities during the 10-month follow-up period. One patient experienced tumor progression, and overall survival was 8-months (2–19-months). Mori et al. reported two IDEM metastases of lung origin. Both spinal cord lesions were in a previously irradiated field, receiving up to 42 Gy for either the lung primary lesion (via standard fractionated external beam radiation therapy) or cerebellar metastases (via hypofractionated whole-brain radiation therapy). In both of these patients, IDEM metastases were treated using 25 Gy in either 5 or 10 fractions, relieving neurological symptoms, and controlling tumor growth until death from systemic disease (25).

Veeravagu et al. (26) reported the largest series (*n* = 9) of IM spinal cord metastases treated using SRT. Lesions most commonly arose from breast and lung, though metastases from an epithelioid hemangioepithelioma and teratoma were also included in this series. A median dose of 21 Gy (range 14–27 Gy) was delivered in 1–5 fractions, providing a maximum dose (Dmax) of up to 37.0 Gy (median 26.7 Gy). A particularly large metastasis from inflammatory breast cancer was treated less aggressively in order to minimize the biologically equivalent dose (BED) delivered to the spinal cord and risk of myelopathy. All patients with follow-up were noted to have stable or improved lesions. Though the median overall survival was poor at 4.1-months (range 1–9-months), none of the patients were noted to have radiation-induced toxicity.

Other studies demonstrate similarly low rates of toxicity, despite high dose delivery. Case reports examining the use of SRT or SRS for treating metastases from renal (27), prostate (28), and breast (29) cancers suggest effective tumor control and lack of radiation-toxicity. In Parikh et al. (27) treatment was direct to a cervical (C5) IM metastasis and the patient did report new tinnitus and balance difficulties during the 26-month follow-up after receiving a maximum dose of 18.75 Gy, though this was not explicitly attributed to treatment and the patient also had a history of craniotomy for a temporal lobe metastasis. Lieberson et al. (28) reported effective tumor control following treatment to the conus medullaris with no new neurological deficits at the 3-month follow-up after SRT to the resection cavity with a marginal dose of 27 Gy. Shin et al. (24) reported six IM lesions (five cervical, one thoracic) treated with a single fraction of SRS to 10–16 Gy (median 14Gy). Despite mixed radiographic responses, all patients improved clinically and none had radiation-induced complications reported during the 10-month follow-up.

### Primary Tumors of the Spinal Cord

Spinal cord neoplasms account for 2–4% of all CNS primary tumors (30). The vast majority of adult primary spinal canal tumors are IDEM (80%) and are most commonly diagnosed as meningiomas or nerve sheath tumors (NSTs), which include schwannomas and neurofibromas (NFs) (31, 32). IM tumors account for 5–10% of tumors within the spinal canal, though estimates are higher in children (33). The most common IM tumors are gliomas, which constitute 80–90% of IM primaries, and are predominately ependymomas in adults and astrocytomas in children. Hemangioblastomas (HBs) make up 8–15% of IM lesions. One-third of patients diagnosed with HB have von Hippel-Lindau syndrome, a hereditary condition associated with tumors arising in multiple organs (34, 35). Given the non-infiltrative nature of many spinal tumors, microsurgical resection remains the preferred treatment option. In cases where open surgical resection is contraindicated, stereotactic radiation has been shown to offer symptomatic relief and tumor control. Unlike its use in metastatic lesions, the utility of stereotactic radiation is better characterized for the treatment of primary spinal cord tumors and treatment-related complications have been reported in this context, possibly as a result of the increased number of reports and longer patient survival (Table 2).

**Table 2:** Studies using SRS/SRT for primary tumors of the intradural-extramedullary and intramedullary spinal cord.

**References**	**No. of pts/No. of lesions**	**No. of IDEM/No. of IM**	**Histology**	**Treatment details**	**F/u (mo)**	**Outcomes**	**Complications**
Selch et al. (36)	6/7	–	NSTs (7)	12 Gy in 1 frac	24 (12–42)	One case of transient pain increase	None
						72% lesions stable, 28% responded	
Marchetti et al. (37)	18/21	21/0	MG (11), SW (9), NF (1)	11 lesions 10–13 Gy in 1 frac; 10 lesions 18.5–25 Gy in 4–6 frac	43 (32-73)	64% significant pain relief	1 grade 3 acute toxicity (resolved after 48h); no late toxicity
						67% lesions stable, 33% responded	
Sahgal et al. (38)	16/19	–	NF (11), chordoma (4), HB (2), MG (2)	25 mo (2–37)	25 (2–37)	Partial improvement in pre-treatment pain (15% worsened)	None
						Three tumors progressed	
Dodd et al. (19)	51/55	55/0	SW (30), MG (16), NF (9)	19 Gy (16–30 Gy) in 1–3 frac	36 (24–73)	70% and 50% pain relief in meningioma or schwannomas, respectively	One case of posterior column dysfunction 8 mo after SRS
						61% lesions stable, 39% responded	
Gerszten et al. (39)	73/73	73/0	SW (35), NF (25), MG (13)	Median of 22 Gy, 21.3 Gy, 21.25 Gy to SW, NFs, and MG, respectively	37 (8–71)	Significant pain relief in 14/17 SW and 8/13 NFs	3 cases of Brown-Sequard syndrome at 5, 12, 13 mo after SRS
						100% control rate (stable or responded)	
Chang et al. (40)	20/30	22/8	NSTs (20), HB (8), MG (2)	14–33 Gy in 1–5 frac	35.6 (12–84)	95% pain relief	None
						33% stable, 57% responded, 10% progressed	
Pan et al. (41)	28/46	–	HB (46)	21.6 Gy (15–35 Gy) in 1.8 frac (1–5)	54.3 (3–157)	81.2% symptom improvement	None
						1-year local control rate 96.1%	
Ryu et al. (42)	7/10	0/10	HB (7), ependymoma (3)	21 Gy (18–25 Gy) in 1–3 frac	8 (1–24)	No deterioration of symptoms	None
						70% stable, 30% responded	
Selch et al. (43)	9/20	18/2	HB (20)	12 Gy (12–14 Gy) in 1 frac	51 (14–86)	Symptoms improved in 1/7 tumors	None
						85% stable, 5% responded	
Daly et al. (44)	19/27	7/20	HB (27)	17 lesions 18–30 Gy in 1 frac; 10 lesions 18–25 Gy in 2–4 frac	33.7 (6.6–84)	3-years local control rate 86%	1 grade 2 and 2 grade 1 toxicities at 5, 18, and 26 mo after SRS
Shi et al. (45)	6/10	0/10	Ependymoma (10)	19 Gy (6–24 Gy) in 1–3 frac	54 (2–157)	2-years local control rate 100%	None

*Lesions are intradural unless otherwise specified as IDEM or IM. IDEM, intradural-extramedullary; IM, intramedullary; f/u, follow-up; frac, fractions; mo, months; NF, neurofibroma; HB, hemangioblastoma; MG, meningioma; SW, schwannoma; NST, nerve sheath tumor*.

Several large series of IDEM tumors treated by SRT or SRS have been published and describe a small number of transient treatment-related complications. Selch et al. (36) reported the results of 25 benign NSTs, including seven intradural lesions, treated to 12 Gy in a single fraction. Two of the patients in this series experienced transient worsening of pre-treatment neuropathies following SRS, one of whom had an intradural NST at the cervical level. Both instances of morbidity resolved with conservative treatment, and no patients were documented with long-term spinal cord injury after radiosurgery. In all cases, SRS provided effective tumor local control. Marchetti et al. (37) reported one patient who developed acute worsening of a preexisting sciatica after treatment, though no late toxicity was observed among 18 patients with a median follow up of 38-months. All lesions were reportedly stable or decreased on radiological follow-up. In a series of a similar size, Sahgal et al. (38) treated 19 IDEM lesions to a median of 21 Gy (range 10–30 Gy) in 1–5 fractions. The authors noted that the maximum dose within the tumor volume was not restricted, leading to a median of maximum doses of 26.7 Gy (range 15.4–59.7 Gy). No toxicity was observed during a median follow-up period of 25-months (range 2–37-months) and the majority of patients experienced stable or improvement in pre-treatment pain.

A few studies have reported myelopathy after SRT for a spinal IDEM primary tumor. Dodd et al. (19) treated 55 benign IDEM tumors to 16–30 Gy in 1–5 consecutive days. Despite excellent local control, one patient developed posterior column dysfunction 8-months after radiation, attributed to treatment-related radiation myelopathy. This patient was administered 24 Gy in three sessions for a cervico-thoracic meningioma. The authors note that even though the volume of the irradiated spinal cord was higher in this patient, with over 1.7 cubic centimeters of spinal cord irradiated to over 18 Gy in three sessions, this patient did not undergo a particularly aggressive treatment. Furthermore, this patient underwent two prior surgical resections that may have contributed to his spinal cord injury.

In addition, Gerszten et al. (39) described 3 IDEM lesions out of 73 for which SRS led to radiation-induced myelitis. In three patients, the onset of symptoms was consistent with Brown-Sequard syndrome and occurred at 5–13-months after treatment. All three lesions were treated to 20 Gy in a single fraction at the cervical level. Though none of the affected patients had a history of irradiation, two patients had undergone previous open surgical resection. All tumors were well-controlled following SRS, despite 26% of lesions having recurred after a previous surgical resection. It is interesting to note that the four cases of radiation-myelopathy described here all occurred at the cervical-thoracic level or higher; though, this finding should be weighed against the fact that some of the most common IDEM tumors (NSTs) tend to arise from the cervical region (31).

While hemangioblastomas make a much smaller fraction of IM primary tumors and are only 3% of all spinal neoplasms, the role of SRT in treating HBs is well-documented in comparison to other more common IM tumor histologies (42). This over-representation may be due to its similar vasculature nature to arteriovenous malformations (AVMs), which are commonly treated with SRT and SRS (46, 47).

Treatment of HBs with SRT has been reported in a number of cases. Ryu et al. (42) treated 7 HBs to 21 Gy (range 21–25 Gy), demonstrating stable or improved radiographic and clinical outcomes. Chang et al. (40) found that of eight HBs, six showed sized reduction after treatment to a maximum point dose of 14.5 Gy. In a series of 46 spinal cord HBs, Pan et al. (41) showed that 81.2% of tumors had symptomatic improvement, and actuarial local control rates at 5-years were 92.3%. Prescribed doses ranged from 15 to 35 Gy and, despite a maximum point dose of up to 42.7 Gy, no complications related to SRT were noted. Daly et al. (44) reported a similar efficacy, with a 3-years control rate of 86% among 27 HBs (20 were intramedullary HBs). One grade 2 toxicity was noted 5-months after treatment. This lesion was treated to a maximum cord dose of 17.8 Gy, the lowest among all single-fraction treatments in the series. Of note, this patient had a history of microsurgical resection and SRS 2-years prior to the treatment of interest.

Ependymomas make up the majority of IM tumors in adults, but published reports of IM ependymoma tumors treated using SRS are rare (32). Ryu et al. treated three IM ependymomas to 18 Gy in one or two sessions. All three lesions had improved clinical outcomes at follow-up (range 1–12-months) (42). In a recent series of intracranial and spinal ependymomas, Shi et al. (45) described 10 spinal cord ependymomas treated using SRT to a median of 19 Gy. Interestingly, half of the patients (including intracranial lesions) were pediatric cases of ependymoma, though it is unclear how many of those were spinal lesions. No radiation-related injuries were reported during the follow-up period (median 54-months, range 2–157-months).

## Discussion

Preliminary evidence suggests that SRT and SRS are effective and safe for treating lesions of the intradural and intramedullary space. With advances in microsurgical technique, resection remains the preferred choice for both primary and metastatic lesions of the spinal cord. Excluding a small number of tumor types, including primary IM spinal cord lymphomas and some pediatric malignancies, pharmacologic treatment have a limited role in primary IDEM and IM lesions (8, 48). Among spinal cord metastatic lesions with systemic disease that may respond chemotherapeutic agents, most studies have demonstrated a lack of survival benefit (49–51). As the utility of chemotherapy is made on a case-by-case basis and largely limited by the tumor histology, surgery and radiation are more applicable treatment options. In cases where the lesion is not amenable to surgery, or if residual tumor remains following resection, stereotactic radiation can provide a valuable treatment. Though the risk of radiation-induced injury exists, the appropriate treatment plan must be weighed against the expected prognosis of the patient's disease.

Assessing the risk of radiation-injury when treating IDEM and IM metastases with SRT is challenged by the short overall survival portended by aggressive systemic disease and possible CSF seeding. Brain metastases are reported to occur in two-thirds of IDEM metastases, suggesting that the CSF pathway plays an important dissemination point in the pathogenesis of IDEM metastases (52). The largest series of IDEM metastases reported by Shin et al. (24) note brain metastases in all patients, and many of the studies cited in this review also note short follow-ups due to the underlying malignancies. Given that the incidence curve of post-treatment neuropathy flattens after 15-months, the risk of spinal injury in this population may be underestimated by existing literature (15). However, this risk should be weighed against the already-demonstrated benefits of treating metastases with SRT, including symptomatic improvement and pain relief (24–28).

Among primary spinal lesions, the majority of which are benign slow-growing tumors, the long-term consequences of spinal injury may have greater impact due to prolonged life expectancies. These tumors tend to be non-infiltrative and amenable to total tumor resection, yielding low recurrence rates when gross total removal is achieved (3, 53, 54). In published series for primary spinal tumors, SRT has been demonstrated to yield excellent local control in nerve sheath tumors, meningiomas, and hemangioblastomas (19, 38, 40–42, 44, 55). Given that induced radiation-injury would drastically decrease quality of life over an expectedly long life-expectancy, understanding factors that influence radiation-induced injury is crucial to identifying patients who would be good candidates for SRT.

Though several guidelines have been published for treating spine tumors using SRT, the accepted tolerance for radiation to the spinal cord remains debated. The upper limit of 10 Gy for maximum dose is commonly cited to be yield safe results with no radiation-related complications, though estimates range from 8 to 13 Gy (56, 57). Among existing published reports, the radiation dose delivered to a particular lesion is often greater than commonly cited upper-bounds. Furthermore, incidences of radiation-injury are not necessarily correlated with the most aggressive SRT treatment plan. Understanding the risk profile of radiation doses is important as many groups will take special care to limit the maximum point dose, potentially reducing the efficacy of SRT in the context of radiation dose-dependent tumor growth response (58).

The risk of injury from radiation therapy is likely multifactorial (59, 60). It has been speculated that prior open surgeries may predispose the spinal cord to subsequent radiation injury (39). Animal models showed that 2-years in between full-dose radiation treatments to the spinal cord is sufficient for recovery, raising additional concerns about how more recent prior radiotherapy affects radiation-injury after SRT in humans (61). Bijl et al. (62) showed that, contrary to assumptions that probability of myelopathy is proportional to length of irradiated spinal cord, damage to the spinal cord decreases when the field length is reduced. A separate study demonstrated an increase in the partial cord tolerance compared to full-thickness cord irradiation (63). Other animal studies have suggested location along the spinal cord can also affect radiation sensitivity, as the thoracic spine may be more prone to injury due to its poor vascular supply (64). Even within the same anatomical level, some studies have suggested variations in radio-sensitivity between white and gray matter (63). While many of these conclusions are drawn from experiments in animal models, the role of these factors are not well-elucidated in humans. Understanding what factors influence stereotactic radiation-related injury is likely oversimplified by the existing emphasis on maximum tumor dose. Larger studies with detailed dosimetry data, lesion characteristics, and treatment history are needed to understand what factors contribute to radiation-induced injury.

## Conclusion

SRT provides effective tumor control and symptomatic relief in primary and metastatic spinal cord lesions. Acute and delayed incidences of treatment-related radiation-injury have been reported. While this review is insufficient for determining the tolerance of the spinal cord against injury, it suggests that high maximum dose is not necessarily correlated with treatment injury. Larger studies with longer follow-up are needed to assess whether and how histology, prior treatments, anatomical level, and the radiosurgery treatment plan affect the risk of treatment-related radiation myelopathy.

## Author Contributions

EL wrote the manuscript. JS and ES formulated project and edited manuscript. All authors contributed to the manuscript revision, read, and approved the submitted version.

## Conflict of Interest

The authors declare that the research was conducted in the absence of any commercial or financial relationships that could be construed as a potential conflict of interest.
